# Dynamic rehabilitation needs and quality of life in postoperative glioma patients

**DOI:** 10.1097/MD.0000000000045915

**Published:** 2025-11-14

**Authors:** Anna Guo, Juhua Li, Huiyun Wang, Qin Xu

**Affiliations:** a Department of Neurosurgery, Jiangsu Province Hospital and the First Affiliated Hospital of Nanjing Medical University, Nanjing, PR China; b School of Nursing, Nanjing Medical University, Nanjing, PR China.

**Keywords:** dynamic changes, glioma, postoperative care, quality of life, rehabilitation needs

## Abstract

**Background:**

Postoperative glioma patients experience diverse rehabilitation needs impacting their quality of life (QoL). Understanding the dynamic changes in these needs and their relationship with QoL is crucial for developing tailored care strategies.

**Methods:**

Using convenience sampling, 115 postoperative glioma patients from the neurosurgery department of The First Affiliated Hospital of Nanjing Medical University were enrolled. Rehabilitation needs and QoL were assessed using standardized questionnaires at 3 postoperative phases: T1 (early), T2 (3 months), and T3 (6 months).

**Results:**

Rehabilitation needs were significantly influenced by age, clinical stage, education, surgery type, disease knowledge, and monthly household income (*P* <.05). Total needs scores and specific dimensions varied significantly across T1, T2, and T3 (*P* <.05): Physiological, spiritual, and psychological needs decreased by 3 months (T2). Care and information needs declined by 6 months (T3).Self-esteem needs increased progressively, peaking at T3. A negative correlation existed between rehabilitation needs and QoL at all time points (*P* <.05), indicating higher needs corresponded to poorer QoL.

**Conclusion:**

Postoperative glioma patients exhibit dynamic, multifaceted rehabilitation needs influenced by clinical and socioeconomic factors. The 3-month postoperative phase (T2) represents a critical window for intervention, characterized by heightened needs and diminished QoL. The consistent inverse relationship between needs and QoL underscores the necessity for personalized, phase-specific rehabilitation programs to optimize patient outcomes.

## 1. Introduction

Over the past 3 decades, there has been a notable increase in the incidence of malignant central nervous system tumors. Recent global statistics highlight China, the United States, and India as the top 3 countries with the highest morbidity and mortality rates attributed to malignant brain tumors.^[1,2]^ Neurogliomas represent the predominant subtype among malignant cranial tumors within the adult central nervous system, comprising approximately 75% of all primary malignant cranial tumors. Their incidence is around 5.26 cases per 100,000 individuals.^[3,4]^ According to the recent Cancer Statistics Report,^[5]^ With 23,890 cases of cranial and other neurological tumors diagnosed in the United States in 2022 and approximately 18,020 deaths, this is now the leading cause of cancer deaths in men under 39 years of age and women under 19 years of age. Neurogliomas are highly malignant and have an extremely high mortality rate and a high risk of recurrence, placing a significant burden on society and families.

Clinical symptoms in patients with glioma include headaches and focal neurological symptoms such as paresis, visual perceptual deficits, sensory loss, and seizures.^[6]^ In addition, the diagnosis and treatment of malignant tumors are coupled with an increased disease burden due to various neurological and cognitive symptoms. Surgery is currently the preferred treatment strategy for gliomas, leading to precise histological diagnosis, tumor genotyping, and improving patient prognosis. A review of the literature found that 56% to 88% of glioma patients experience psychoneurological symptoms such as anxiety, depression, sleep disturbances, fatigue, and pain after surgery.^[7]^ These symptoms often occur together and are synergistically reinforcing.^[8]^ The patient’s physical and mental health and quality of life can be somewhat affected.

From the time of cancer diagnosis to surgical treatment and subsequent adjuvant radiotherapy, patients have various rehabilitation needs in terms of physical, psychological, and social support. The study shows that about 80 percent of cancer patients have unmet rehabilitation needs.^[9]^ Meeting the needs of patients’ rehabilitation requirements is the basis for the sustainable development of nursing, and the fundamental goal of improving the quality of nursing services is also an essential responsibility of every nursing worker. Especially in the 21st century, the rehabilitation needs of oncology patients are diversified in terms of content and form, and they need to be understood, cared for, and respected in addition to the requirements of superior medical skills and excellent services. In particular, patients with gliomas may suffer from headaches, vomiting, seizures, disturbance of consciousness, abnormal behaviors, intellectual decline, and neurological deficits because of intracranial occupying effects and increased intracranial pressure. Symptoms are even sometimes mistaken for a series of problems such as senile dementia or psychiatric disorders.^[10]^ Therefore, meeting the comprehensive rehabilitation needs of glioma patients in various aspects of society, physiology, psychology, and life is significant in promoting disease recovery and improving the quality of life. An extensive literature search has revealed that not much research has been done in China on the rehabilitation needs of gliomas, and the direction of research has been limited. Foreign scholar Using Maslow hierarchy of needs theory to investigate the needs of postoperative glioma patients, we investigated the psychological, physiological, and social aspects of the patient’s needs separately and suggested that age, family structure, and the number of surgeries were all critical factors affecting the patients’ needs.^[11]^ Rossi et al argued that a few healthcare professionals could not meet all the needs of oncology patients and that volunteers or healthcare professionals should be mobilized to create a social support group to promote patients’ recovery.^[12]^ Stensvold et al surveyed glioma patients, noting that patients’ needs for health education focused on several aspects of surgical methods, outcomes, complication management, and psychological support and counseling.^[13]^

The current situation and changes in the rehabilitation needs of tumor patients have been explored in China. Most of them focus on cross-sectional studies, with fewer longitudinal studies and even fewer studies related to the rehabilitation needs of patients with gliomas, which do not provide a complete picture of the rehabilitation needs of the patients at different times of the disease. Given this, the study adopts a longitudinal approach to understand the changing trend of rehabilitation needs of glioma patients and the correlation with their quality of life to guide clinical healthcare professionals to provide patient-need-oriented and more targeted care services for glioma patients and improve their quality of life.

## 2. Methods

### 2.1. Research object

Patients with glioma admitted to our neurosurgery department from November 2022 to November 2023 were selected using convenience sampling. Inclusion criteria: histologically diagnosed glioma; age > 18 years; Consciousness and regular expression; voluntary participation in this study. Exclusion criteria: comorbid history of other severe systemic diseases; recent history of antipsychotic medication; mental illness or cognitive impairment. Exclusion criteria: patients who withdrew from the study in the middle of the study or died. According to the rough estimation method, the sample size is 5 to 10 times the number of variables. Fifteen variables were included in this survey, so the required sample size was 75 to 150 cases, and considering the 15% failure rate, it should be 86 to 173 cases; 120 research subjects were selected for this study, and 115 cases were included in the adequate sample.

### 2.2. Research tools

The subject group prepared general patient information, including socio-demographic and clinical information. Socio-demographic data included age, gender, marital status, education level, economic income, and type of health insurance; clinical data included disease duration, disease awareness, comorbidities, treatment modality, cancer stage, and tumor location.

Rehabilitation Needs Scale This scale was developed by Zhao Lingzhu et al in 2019, based on the guidance of Maslow hierarchy of needs theory, and is mainly used to assess the rehabilitation needs of neurological patients.^[14]^ The scale consists of 5 dimensions, namely, physiological, spiritual, psychological, self-esteem, information, and care needs, with 28 entries. Each entry is scored on a 5-point Likert scale from “not important” to “very important,” with a 1 to 5. Each entry is positively scored; the higher the score, the higher the patient’s rehabilitation needs. Its content validity index (S-CVI) was 0.960, the overall Cronbach alpha coefficient of the scale was 0.731, and the dimensions’ alpha coefficients ranged from 0.732 to 0.795. The scale can assess the rehabilitation needs of patients with different types of neurological diseases with good reliability and validity.

The European Organization for Research and Treatment of Cancer’s core Quality of Life questionnaire (EORTC QLQ-C30) assesses the quality of life in multiple areas of brain tumor patients.^[15]^ It consists of 5 functional dimensions (physiological, role, emotional, cognitive, and social functions), comprising 30 entries. In particular, entries 29 and 30 were divided into 7 levels, which were counted as 1 to 7 depending on their response options; the other entries were divided into 4 levels: from none, a little, and more to a lot, and when scoring, they were rated directly from 1 to 4. The Cronbach alpha number for the scale in this study was 0.94.

### 2.3. Data collection

This study protocol has been reviewed and approved by the Ethics Committee of the First Affiliated Hospital of Nanjing Medical University, with the ethical approval number: 2023-SR-443. The study began with a pre-survey of 10 randomly selected cases that met the inclusion criteria. After the questionnaire was perfected, a targeted questionnaire research was carried out on 120 cases again, with the main instruments of the study being the General Information Questionnaire, the Needs evaluation questionnaire (The Needs evaluation Questionnaire, NEQ), and the Quality of Life Inventory. According to the 8th edition of Surgery, patients with glioma should be reviewed every 3 months for 1 year after treatment, and in this study, the measurements were taken 3 times. The data were collected at 7 days after surgery (T1), 3 months after surgery (T2), and 6 months after surgery (T3). Before the survey, the researchers obtained approval from the head of the hospital department, conducted full communication and coordination to gain support, and received unified training on the purpose, content, and precautions of the study. After screening patients according to the inclusion and exclusion criteria, eligible patients were fully informed of the study purpose, methods, potential risks, and rights. The survey was only initiated after obtaining their voluntary informed consent and having them sign the Informed Consent Form. On-site surveys were conducted using a combination of semistructured interviews and self-completion by patients, with a commitment to strict confidentiality of patient information. A total of 120 questionnaires were distributed on-site 7 days after surgery, and 120 valid questionnaires were returned, resulting in a recovery rate of 100%. Follow-ups at 3 and 6 months after surgery were conducted through a combination of offline face-to-face surveys, online emails, and telephone surveys. At 3 months postoperatively, 2 patients refused to cooperate with the survey due to poor mood and physical function. At 6 months postoperatively, 3 patients died and could not complete the survey. Eventually, 115 patients completed the survey at all 3 time points.

### 2.4. Quality control

Before the survey: uniform training for researchers, uniform guidelines, familiarization with the questionnaire content, interpretation of questionnaire entries, and questionnaire review criteria to ensure that the survey methodology is consistent, the data obtained is accurate and reliable, to avoid measurement bias, and to conduct a pre-survey before the formal survey.

In the survey, the researcher distributed questionnaires one-to-one, recovered and checked the questionnaires, and asked the research participants to complete them promptly if any items were missing or omitted.

Post-survey: Data entry is carried out in pairs, and data are checked individually for authenticity and accuracy.

### 2.5. Statistical analysis

The data were statistically analyzed using SPSS 26.0 software (Chicago): data such as percentages and frequencies were used to statistically describe the general information and rehabilitation needs of the sample of this study. Multiple linear regression was used, leading to an in-depth analysis of the factors influencing the demand for rehabilitation regarding glioma patients. Repeated-measures analysis of variance (ANOVA) was used, and 2-by-two comparisons were made using the least significant difference method to compare the differences in the postoperative rehabilitation needs of patients with glioma at different stages of the disease. Pearson correlation analysis was used to explore the correlation between rehabilitation needs and quality of life of glioma patients at different time points.

## 3. Results

### 3.1. General information for patients with glioma

115 patients with glioma, aged 18 to 92 years, with a mean of (61.25 ± 15.27) years, were investigated in this study, and the rest of the data are shown in Table 1.

**Table 1:** General information on patients with glioma.

Item	Subgroup	Number of cases	Composition ratio (%)
Age	18–40 yr old	40	34.8
	41–59 yr old	58	50.4
	≥60 yr old	17	14.8
Sex	Male	60	52.2
	Female	55	47.8
Marital status	Married	80	69.6
	Unmarried	35	30.4
Educational attainment	Primary school and below	27	23.5
	Junior and senior high school	60	52.2
	Bachelor’s degree or above	28	23.5
Economic Income (RMB/month)	<1000	2	1.7
	1000–3000	27	23.5
	3000–5000	45	39.1
	5000–10,000	35	30.4
	>10,000	6	5.2
Type of medical insurance	self-financed	5	4.3
	Medical insurance publicly	100	87.0
	funded medical care	10	8.7
Disease duration	<6 mo	85	73.9
	6 mo–1 yr	18	15.7
	>1 yr	12	10.4
Disease awareness	I don’t know	38	33.0
	Understood	50	43.5
	Familiar	27	23.5
Comorbidity	No	25	21.7
	High blood pressure	50	43.5
	Diabetes	40	34.8
Treatment	Surgery	51	44.3
	Surgery + chemotherapy	39	33.9
	Surgery + chemotherapy + radiotherapy	25	21.7
Clinical Staging	I	80	69.6
	II	18	15.6
	III	15	13.0
	IV	2	1.7
Tumor location	Frontal lobe	65	56.5
	Temporal lobe	18	15.7
	Parietal lobe	14	12.2
	Occipital lobe	10	8.7
	Others	8	7.0

### 3.2. Analysis of factors influencing the rehabilitation needs of glioma patients

Statistical analysis of the rehabilitation needs of glioma patients with different age, gender, marital status, and other information showed statistically significant differences in the overall scores of patients’ age, education, economic income, knowledge of the disease, treatment modalities, and clinical staging (*P* < .05), as shown in Table 2.

**Table 2:** Comparison of rehabilitation needs scores of glioma patients with different profiles.

Item	Subgroup	Number of cases	Rehabilitation needs score ($\bar{x}\pm \;s$)	*F*/*t*	*P*
Age	18~40 years old	40	123.02 ± 35.71	7.123	<.001
	41–59 years old	58	112.54 ± 28.43		
	≥60 years old	17	89.37 ± 25.63		
Educational attainment	Primary school and below	27	120.65 ± 39.32	3.545	<.005
	Junior high school and high school	60	107.98 ± 31.66		
	Undergraduate and above	28	90.29 ± 29.97		
Economic Income	<1000	2	131.83 ± 40.21	2.092	<.005
	1000~3000	27	123.67 ± 37.98		
	3000~5000	45	110.56 ± 35.32		
	5000~10,000	35	102.22 ± 41.28		
	>10,000	6	89.24 ± 29.66		
(Yuan/month)	I don’t know.	38	126.45 ± 29.76	3.986	<.005
	Understood	50	114.07 ± 32.48		
	Familiar	27	97.01 ± 27.84		
Disease awareness	Surgery	51	108.54 ± 37.79	6.176	<.001
	Surgery + Chemotherapy	39	115.45 ± 40.09		
	Surgery + chemotherapy + radiotherapy	25	128.09 ± 31.45		
Treatment	I	80	96.67 ± 23.56	5.877	<.001
	II	18	103.06 ± 30.56		
	III	15	112.57 ± 39.03		
	IV	2	124.65 ± 38.91		

### 3.3. Multiple linear regression analysis of factors influencing rehabilitation needs of postoperative glioma patients

Based on the multiple linear regression analysis, it can be concluded that the respective variables influence the demand for rehabilitation in descending order: age, literacy, clinical stage, treatment modality, disease awareness, and economic income, as shown in Table 3.

**Table 3:** Multiple linear regression analyses of factors influencing patients’ rehabilitation requisites after glioma surgery.

Dependent Variable	Bias regression coefficient	Standard Error	Standard regression coefficient	*t*	*P*
constant	112.452	14.56	–	10.435	<.001
Age	−18.467	3.654	−0.438	−4.821	<.001
Educational attainment	−5.452	2.076	−0.256	−2.812	.007
Economic Income	−0.765	2.245	−0.032	−0.373	.722
Disease awareness	−7.775	3.076	−0.224	−2.188	.029
Clinical Stage	8.991	3.227	0.264	2.543	.01
Treatment	5.543	3.098	0.132	2.394	.018

R2 = 0.332, adjusted R^2^ = 0.314, F = 8.776, *P* < .01; – refers to the blank term.

### 3.4. Trends in dimensions and total score of rehabilitation needs of glioma patients

The results of the comparative analysis of the rehabilitation need scores of postoperative glioma patients at 7 days postoperatively, 3 months, and 6 months using repeated-measures ANOVA were: the changes in the total rehabilitation need scores and scores of the dimensions at each node were statistically significant (*P* < .05), as shown in Table 4.

**Table 4:** Trends in dimensions and total score of rehabilitation needs of glioma patients.

Variables	T1	T2	T3	*F*	*P*
Physiological needs	2.432 ± 0.921	4.028 ± 1.144	2.266 ± 1.044	68.554	<.001
Spiritual and psychological needs	2.912 ± 1.117	3.677 ± 1.226	2.448 ± 0.872	67.331	<.001
Self-esteem needs	2.433 ± 0.778	2.855 ± 1.071	3.154 ± 0.672	38.213	<.001
Information needs	3.535 ± 1.042	2.774 ± 0.779	2.164 ± 0.705	79.446	<.001
Care needs	3.642 ± 1.209	2.786 ± 0.572	2.013 ± 1.025	100.332	<.001
Total score	3.144 ± 0.885	3.311 ± 0.522	2.397 ± 0.609	102.125	<.001

### 3.5. Correlations between dimensions of rehabilitation needs and total scores and quality of life at different time points in glioma patients

Correlation of dimensions and total scores of rehabilitation needs with quality of life at the T1 time point in glioma patients Pearson analysis revealed a strong association between rehabilitation needs and quality of life scores at the T1 node. The total rehabilitation needs score and the specific scores of each dimension were negatively associated with the total quality of life score (*P* < .01), as shown in Table 5.

**Table 5:** Rehabilitation needs and quality of life correlations in patients with T1 node gliomas.

Variables	Physiological needs	Spiritual psychological needs	Self-esteem needs	Information needs	Care needs	Rehabilitation needs total score
Physiological needs	1	–	–	–	–	–
Spiritual and psychological needs	0.564*	1	–	–	–	–
Self-esteem needs	−0.035	0.189**	1	–	–	–
Information needs	−0.031	0.081	0.230*	1	–	–
Care needs	−0.411*	−0.543*	−0.258*	0.025	1	–
Rehabilitation needs total score	0.509*	0.806*	0.414*	0.336*	0.805*	1
Total quality of life score	0.154	−0.142	−0.276*	−0.321*	−0.407*	−0.510*

* *P* <.01.

** *P* <.05.

Correlation of dimensions and total scores of rehabilitation needs with quality of life at T2 time point in glioma patients The results showed that the rehabilitation needs of postoperative glioma patients at 3 months postoperatively (T2) were significantly and negatively associated with the total quality of life scores for all dimensions and total scores except for the physiological needs dimension; among them, the total scores of self-esteem needs, information needs, and caregiving needs were negatively correlated with the total quality of life scores (*P* < .01), as shown in Table 6.

**Table 6:** Correlation between rehabilitation needs and quality of life in patients with T2 node glioma.

Variables	Physical needs	Spiritual and psychological needs	Self-esteem needs	Information needs	Care needs	Rehabilitation needs total score
Physiological needs	1	−	−	−	−	−
Spiritual and psychological needs	0.332*	1	−	−	−	−
Self-esteem needs	−0.093	0.228**	1	−	−	−
Information needs	−0.134	0.017	0.118	1	−	−
Care needs	−0.234*	−0.168**	−0.544	0.025	1	−
Rehabilitation needs total score	0.311*	0.603*	0.342*	0.336*	0.437*	1
Total quality of life score	0.154	−0.142	−0.276*	−0.019	−0.441*	−0.377*

* *P* <.01.

** *P* <.05.

Correlation of dimensions and total scores of rehabilitation needs with quality of life at T3 time point in glioma patients In the final analysis, T3 and T2 nodes were the same, and all the dimensions and total scores of rehabilitation needs, except for the physical needs dimension, showed significant negative associations with the total quality of life scores; among these dimensions, self-esteem needs, care needs, and information needs were negatively associated with the total quality of life scores (*P* < .05), see Table 7.

**Table 7:** Correlation between rehabilitation needs and quality of life in patients with T3 node glioma.

Variables	Physiological needs	Spiritual psychological Needs	Self-esteem needs	Information needs	Care needs	Rehabilitation needs total score
Physiological needs	1	–	–	–	–	–
Spiritual and psychological needs	0.162	1	–	–	–	–
Self-esteem needs	−0.128	−0.164	1	–	–	–
Information needs	−0.078	0.277*	−0.057	1	–	–
Care needs	−0.097	0.144	0.189**	0.655*	1	–
Rehabilitation needs total score	0.379*	0.714*	0.165	0.611*	0.679*	1
Total quality of life score	0.096	−0.142	−0.189**	−0.207**	−0.188**	−0.170

* *P* <.01.

** *P* <.05.

## 4. Discussion

### 4.1. Factors influencing the postoperative rehabilitation needs of glioma patients

#### 4.1.1. Patients of different ages have different rehabilitation needs

This study shows that the patient’s age is inversely related to the need for rehabilitation; the younger the patient is, the greater the need for rehabilitation. This is in line with the findings of Paltrinieri et al.,^[16]^ which analyzed the reason for this is that the younger the patient, the more concerned they are about the impact of gliomas on their physical functioning, their family and work, and therefore the younger the patient, the more health information they need. The older the patient, the more tolerant he or she is, the better able he or she is to adapt to the effects of the disease on his or her body, and the better able he or she is to cooperate with the treatment. The population included in this study focuses on young and middle-aged people, who are the pillars of the family and bear the pressure of supporting parents and educating children themselves, in addition to the trouble of glioma disease, which is very likely to cause emotional instability and lead to excessive mental and psychological stress. Postoperative glioma patients may experience image changes such as unsteady walking and slurred speech, and the younger the patients are, the more they pay attention to their image and the higher their self-esteem needs. Therefore, it is suggested that clinical staff should pay attention to the psychological state of young glioma patients after surgery and take the initiative to provide disease-related information to increase patient cooperation and a sense of security.

#### 4.1.2. Educational attainment affects the rehabilitation needs of postoperative glioma patients

Education level is also an essential factor affecting rehabilitation needs. The 2 are negatively proportional, i.e., the lower the patient’s education level, the more rehabilitation needs they have, the same as the findings of Wang et al.^[17]^ Patients with higher education levels have relatively higher levels of information acceptance and understanding of the disease, can better cooperate with the treatment, and can also take the initiative to take corresponding measures to cope with the side effects of the treatment and reduce the uncomfortable symptoms. It is suggested that the influence of education level on patients’ needs should be considered in clinical work. For patients with lower education levels, verbal expression should be used in health education, and at the same time, some easy-to-understand pictures can be added. More network information technology can be used for patients with higher education degrees.

#### 4.1.3. Economic income affects the rehabilitation needs of postoperative glioma patients

Economic income also has a particular impact on rehabilitation needs. The present analysis concludes that a relatively high level of per capita income corresponds to a relatively low demand for rehabilitation, which is the same as the results of Lam et al It analyses why the treatment of glioma is a long process, why the treatment cost is high, and why it significantly impacts the economic pressure of patients.^[18]^ Patients with better economic conditions pay more attention to the disease changes and less to the treatment plan. Still, patients with poor economic conditions affect the treatment decision because of the cost of treatment.

#### 4.1.4. The level of disease awareness affects the rehabilitation needs of patients after glioma surgery

This analysis underscores the pivotal role of disease perception in shaping rehabilitation needs, with patients possessing more excellent knowledge of their condition exhibiting reduced reliance on rehabilitation services. Lam et al focused on 3 fundamental dimensions – physical, informational, and caregiving – and found that patients’ understanding of their illness significantly influences their psychological status and coping strategies. Insufficient disease awareness engenders feelings of uncertainty and exacerbates negative psychological states, thereby escalating psychological needs.^[18]^ Left unaddressed, these needs can substantially impact physical functioning, leading to heightened physical and daily living assistance requirements. Moreover, limited disease awareness heightens the need for care and support, heightening expectations for guidance and social assistance. To address these challenges, clinical and professional personnel are encouraged to educate glioma patients on symptom management strategies post-surgery while enhancing postdischarge continuity of care to ensure timely outpatient guidance and support.

#### 4.1.5. Clinical staging and treatment modalities influence the rehabilitation needs of patients after glioma surgery

This study shows that both clinical stage and treatment modality can influence patients’ rehabilitation needs, with the more advanced patients and the more complex the treatment modality, the more unmet rehabilitation needs. It was found that the degree of psychoneurological symptoms,^[19]^ general symptoms, and peripheral nerve symptoms of patients with neuroglioma stage III or higher were more severe than those of patients with stage I and II, indicating that the higher the clinical stage of the patient’s disease, the more complex the treatment modality, and the more severe the symptoms were, the more the patient’s physiological and psychological stress increased, and the higher the need for rehabilitation. Analyzing the reasons, the larger the scope of the surgery, the more pronounced the symptoms of wound pain, discomfort caused by drainage tubes, and fatigue in a shorter period after the surgery. This suggests that clinical staff should respond positively to patients’ discomfort and needs, strengthen communication with patients, and take necessary measures to alleviate pain.

#### 4.1.6. Impact of Tumor Location on Postoperative Rehabilitation Needs in Patients with Glioma

**Figure 1.**
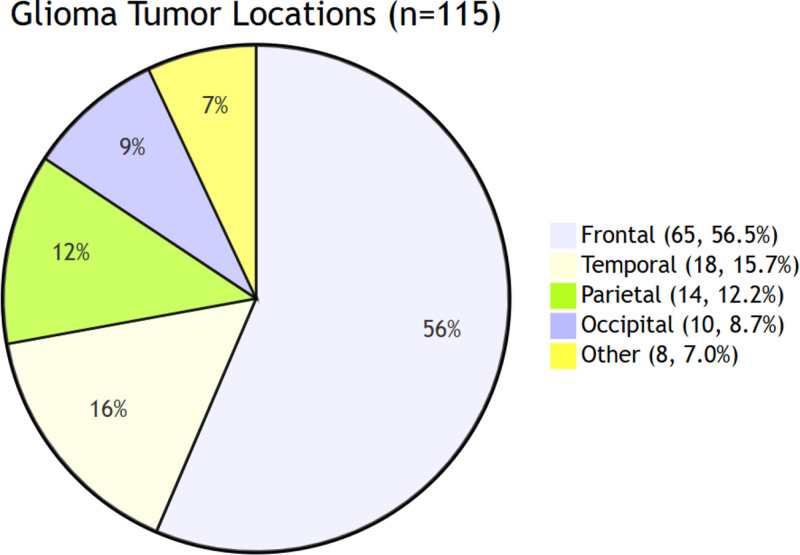
Glioma tumor locations (n = 115).

Although previous studies have indicated that glioma location may significantly influence postoperative rehabilitation needs – such as psychological support for frontal lobe tumors and motor rehabilitation for parietal lobe tumors^[20]^ – the present study did not detect statistically significant differences (*P* > .05). This discrepancy may be attributable to several factors. First, the proportion of frontal lobe tumors in this cohort was relatively high (56.5%), whereas the non-frontal lobe subgroups were comparatively small (temporal lobe 15.7%, parietal lobe 12.2%, etc; Fig. 1), potentially reducing statistical power and limiting the detection of subtle differences. In addition, tumor grade or treatment modality may have confounded the independent effect of tumor location. Despite the absence of significant differences in our dataset, the potential clinical relevance of frontal lobe tumor location should not be overlooked. Previous research has demonstrated that decreased quality of life in patients with frontal lobe gliomas may be associated with executive dysfunction (e.g., impaired planning ability) and psychiatric symptoms (e.g., depression, apathy).^[20]^ Although no statistically significant differences were observed in this study, the mean QoL scores in the frontal lobe group were lower than in other tumor locations, which may be related to the higher prevalence of psychiatric symptoms in frontal lobe tumors. Therefore, it is recommended that clinicians implement enhanced screening for psychiatric symptoms and initiate early psychological interventions in patients with frontal lobe gliomas, while tailoring targeted rehabilitation programs for patients with non-frontal lobe tumors.

### 4.2. Trends in postoperative rehabilitation needs of glioma patients by dimension over time

In this study, 115 postoperative glioma patients’ information needs and care needs scores declined over time; psychosomatic needs scores were highest at 3 months postoperatively and gradually declined to 6 months postoperatively; and self-esteem needs tended to increase at 3 months postoperatively and rose to a maximum at 6 months postoperatively. Burch et al., in a one-year follow-up work on cases of rectal cancer patients, it is known that along with the prolongation of time, there is a gradual decrease in the need for information, etc, and essentially no change for physiologically related maintenance.^[21]^ Olsson et al conducted a longitudinal study of breast cancer patients over 6 months, which showed a slight increase in self-esteem and psychological needs as a function of the stage of treatment but no significant change in care needs during the follow-up period.^[22]^ The reasons for the differences in the study’s final results were considered to be related to the type of cancer investigated and the time of the start of the investigation. The magnitude of the changes in the needs of postoperative glioma patients was inconsistent across the dimensions at 7 days, 3 months, and 6 months postoperatively.

Patients have the highest demand for information within a short period after surgery. Because the diagnosis of cancer is an adverse event, most of the faces of this bad news are overwhelmed. Thus, this stage of the patient, the treatment of the disease, and prognosis-related information is very urgent. This stage of the in-depth investigation found that the patients are most concerned about the results of the pathology within a short time after surgery, which also determines the severity of the disease and the implementation of the treatment plan to have a clear understanding of their condition, so that the need for information increases, this is similar to the findings of Wiedenbein et al.^[23]^ By 6 months postoperatively, the patient’s information needs were reduced to a minimum, Jesus et al analyzed the reason for this is currently the age of the Internet, the patient’s access to knowledge related to the disease is not difficult, in addition to deepening the understanding of the condition through the communication with healthcare professionals. Thus, the patient’s attention to the information about the disease will be reduced.^[24]^ Therefore, in clinical work, it is necessary to pay attention to the information needs of patients in the short time after the operation, improve the knowledge structure of medical personnel, improve the initiative and self-confidence of clinical medical personnel to provide patients with disease-related information and strengthen the awareness of the health education work of the patients, to enhance the patient’s cooperation and active participation in the treatment.

The care needs of the patients in this study were highest within 7 days postoperatively and kept decreasing after that. This is in line with the findings of Faller et al.^[25]^ The patients in this study wanted more attention and support from healthcare professionals in the hospital. The reason for this may be that the patient’s will to survive is powerful at this time, hoping to be able to get the best medical resources and then quickly alleviate the condition, in addition to the short period after the operation of the medical staff to provide information on the disease and emotional care is precisely the patient’s urgent need at this time. In the actual clinical work process, healthcare workers should reasonably and effectively assess the patient’s living environment, economic situation, and other aspects, fully understand the patient’s resources, and find the best time to implement nursing interventions to help patients effectively cope with the treatment of related discomforts.

Patients’ psychological needs were most significant at 3 months postoperatively, reaching a maximum at this time and gradually decreasing after that. This is similar to the results of the Jesus study.^[24]^ Analyzing the reasons, it was found that the diagnosis of cancer is a highly adverse and stressful event for the patient. Most patients find it difficult to cope with this sudden shock, and with the significant traumatic event of surgery, patients are at a loss. Patients are undergoing intense treatment such as chemotherapy or radiotherapy, which, together with the side effects, gradually increases the psychological burden on the patient. As the treatment is completed, the patient’s psychological needs decline to a plateau at 6 months postoperatively. This also suggests that medical personnel should pay attention to the psychological situation of glioma within 3 months after surgery, construct corresponding psychological evaluation mechanisms, adopt corresponding intervention therapies by the actual psychological and emotional changes of patients the first time, and carry out relevant nursing care for patients’ psychology, such as the current laddering psychological intervention, positive thoughts stress reduction group and other psychological intervention programs with noticeable effects, in addition to encouraging patients to communicate with their families, relatives and friends and patients more often, thus effectively reducing the negative emotions of patients such as nervousness and anxiety. In addition, patients are encouraged to communicate more with their families, relatives and friends, and patients’ friends to effectively reduce the negative emotions of patients, such as tension and anxiety. The physiological needs of the patients are highest in the 3 months after the operation, and after that, they keep decreasing. In addition, postoperative pain, abdominal distension, and fatigue also affect the everyday life of the patients to a great extent. Lewandowska and other scholars pointed out in the research process that the most frequent symptoms of cancer patients are fatigue and exhaustion, which have a very negative impact on the patient’s physical functioning and quality of life.^[26]^ Three months after the operation, the patient is in the period of radiotherapy; due to the radiotherapy, the patient experiences nausea and vomiting, intestinal function damage, bone marrow suppression, hair loss, and other side effects; these uncomfortable symptoms make their physiological and daily life needs reach their peak. As time advances, their physiological symptoms are slowly relieved 6 months after surgery, reducing their needs accordingly. It is recommended that medical staff pay more attention to patient’s physiological symptoms and daily life, especially to their postoperative fatigue symptoms and various reactions during radiotherapy. Depending on their physical condition, patients should be encouraged to participate in appropriate household chores and rehabilitation work and be guided to strengthen their nutritional intake and psychological adjustment.

Six months after the operation, most of the patients’ conditions stabilized, and some completed their treatment plans and entered the follow-up period, with some relief from physical discomfort and a better understanding of their conditions and treatment plans. The patients were able to take positive and effective measures to reduce the impact of the disease on them. Therefore, during this phase, the patient’s psychological, information, and physiological and caregiving needs slowly decrease 6 months after surgery. However, self-esteem needs gradually increased at 6 months postoperatively and reached the highest level at 6 months postoperatively. The difference in patients’ self-esteem need dimension scores at different assessment time points was statistically significant, consistent with previous studies’ results. The reason for this analysis is that patients have returned to everyday work and life and are gradually accepting the presence of the disease, a period in which they are more concerned with issues of body image and altered function. Treatment-related to glioma surgery and radiotherapy can bring about different degrees of body intention disorders, such as craniofacial deformities, hair loss and hyperpigmentation after radiotherapy, steroid-related weight gain, and Cushing-like appearance, which have a severe impact on a patient’s self-esteem. The need for self-esteem grows stronger as time passes, and body image does not change. Therefore, 6 months postoperatively is a critical period to change the patients’ impaired self-esteem, suggesting clinicians should mobilize support from family, friends, and the rest of the community to help the patients out of their body imagery dysfunction dilemma.

### 4.3. Correlations between dimensions of rehabilitation needs and quality of life differ in patients at different stages of postoperative glioma surgery

This study showed that multiple dimensions of patients’ rehabilitation needs at different stages were negatively correlated with quality of life, i.e., the higher the patient’s needs, the worse their quality of life. The scores of the dimensions of rehabilitation needs and the total scores were negatively correlated with the patient’s total quality of life scores at 7 days postoperatively. The total rehabilitation needs score, psychological needs, self-esteem needs, caregiving needs, and information needs dimensions were negatively correlated with the total quality of life scores of postoperative glioma patients at 3 and 6 months post-surgery, with a negative correlation between the self-esteem needs dimension and the total quality of life score at 3 months post-surgery. Six months after the end of the surgery, many of the dimensions of self-esteem, caregiving, and information needs were negatively correlated with the total quality of life scores. It indicates that the negative correlation between multiple domains of rehabilitation needs and quality of life becomes less and less significant with time. Therefore, the clinical work should strengthen the comprehensive assessment of the patient’s needs in the 3 months after surgery, from the physiological and many other aspects of the entry to the maximum degree to ensure the practical improvement of the patient’s quality of life. Information needs and self-esteem needs significantly affected patients’ quality of life throughout the consultation process, which is slightly different from the results of O’Sullivan et al., who studied that psychological needs significantly affected patients’ quality of life throughout the consultation process. Considering that most of the subjects in this study were in their middle and young adulthood, body image and functioning are still an essential part of the quality of life, and patients were more concerned about issues such as impaired self-esteem.^[27]^ Satisfying patients’ information needs helps them deal with problems in a positive way, which in turn affects their quality of life. It is recommended that medical staff assess patients’ rehabilitation needs, especially information and self-esteem, when formulating and implementing care plans and providing health education to promote the recovery of patients’ physical and mental health and improve their quality of life.

### 4.4. Limitations

#### 4.4.1. Single-center sample source

This study was conducted exclusively among postoperative glioma patients from a single tertiary hospital in Jiangsu Province, China, with the majority of participants being early-stage or belonging to specific subtypes. The absence of data from other regions and healthcare settings limits the generalizability of the findings. Therefore, whether the results adequately reflect the overall postoperative rehabilitation needs and quality of life of glioma patients warrants further verification through multicenter studies.

#### 4.4.2. Short follow-up period

Although this study employed a longitudinal design, the follow-up duration was limited to 6 months. This relatively short observation period precludes the assessment of long-term changes in rehabilitation needs and quality of life, including the potential effects of persistent functional impairment, psychological adaptation, or tumor recurrence. Future studies should incorporate extended follow-up periods to clarify long-term trajectories.

#### 4.4.3. Incomplete coverage of influencing factors

The rehabilitation needs and quality of life of postoperative glioma patients are shaped by multiple determinants, such as tumor molecular pathology, extent of surgical resection, and family support. In this study, only general demographic and clinical variables were analyzed, leaving potential unmeasured confounders unaccounted for. As such, the generalizability of the conclusions should be interpreted with caution.

## 5. Conclusion

This longitudinal study demonstrates that postoperative glioma patients exhibit dynamic and multifactorial rehabilitation needs, which are significantly influenced by sociodemographic and clinical factors such as age, education, income, disease awareness, clinical stage, and treatment modality. Rehabilitation needs vary across postoperative phases, with the 3-month period emerging as a critical window characterized by heightened physiological, psychological, and self-esteem demands alongside diminished quality of life. The persistent negative correlation between rehabilitation needs and quality of life underscores the necessity for personalized, phase-specific, and multidisciplinary rehabilitation strategies. Tailored interventions that prioritize information provision, psychological support, and self-esteem restoration – particularly during the early and mid-postoperative stages – are essential for optimizing long-term recovery and enhancing patient quality of life.

## Acknowledgments

We thank all the participants of the study.

## Author contributions

**Conceptualization:** Qin Xu.

**Data curation:** Anna Guo, Juhua Li, Huiyun Wang.

**Formal analysis:** Anna Guo.

**Funding acquisition:** Qin Xu.

**Methodology:** Anna Guo, Juhua Li.

**Project administration:** Qin Xu.

**Resources:** Qin Xu.

**Writing – original draft:** Anna Guo.

**Writing – review & editing:** Anna Guo, Juhua Li.
